# P-124. Evolving Epidemiology of Bacterial Central Nervous System Infections at a Large Urban Tertiary Care Hospital: A 25-Year Review

**DOI:** 10.1093/ofid/ofaf695.351

**Published:** 2026-01-11

**Authors:** Lior Cohen Yatziv, Alfredo J Mena Lora, Scott Borgetti

**Affiliations:** University of Illinois Chicago, Chicago, Illinois; University of Illinois Chicago, Chicago, Illinois; University of Illinois at Chicago, Chicago, Illinois

## Abstract

**Background:**

Central nervous system (CNS) infections caused by diverse pathogens are life-threatening and associated with severe neurological sequelae and substantial impacts on healthcare systems worldwide. When CNS infections occur in a healthcare-associated (nosocomial) setting, they are characterized by distinct microbial etiologies. Since the COVID-19 pandemic, antimicrobial resistance (AMR) infections have increased dramatically, notably altering the resistance profiles of nosocomial infections.

**Figure:**
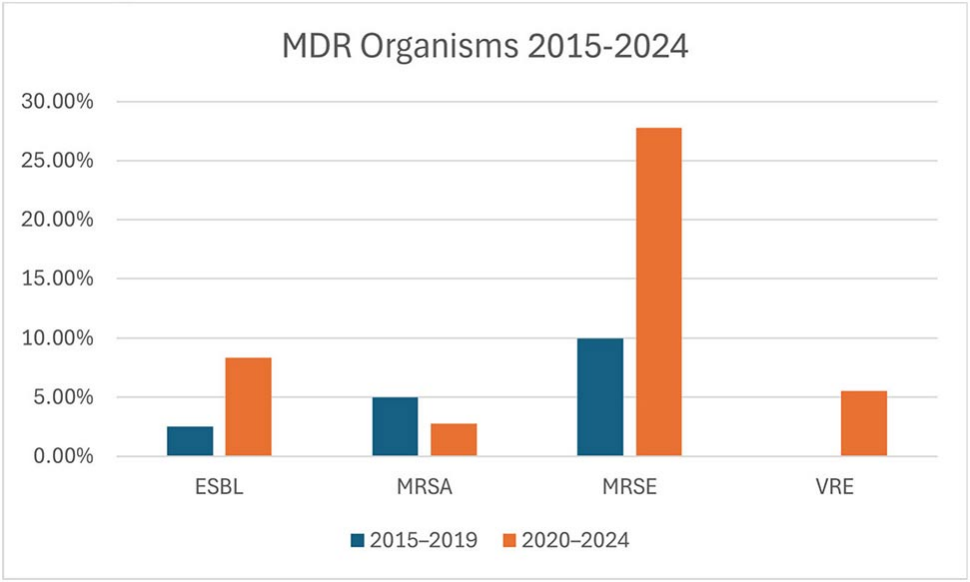
MDR Organisms 2015-2024 MDR - Multidrug resistance, ESBL - Extended-spectrum β-lactamase, MRSA - Methicillin-resistant Staphylococcus aureus, MRSE - Methicillin-resistant Staphylococcus epidermidis. VRE - Methicillin-resistant Staphylococcus epidermidis

**Figure:**
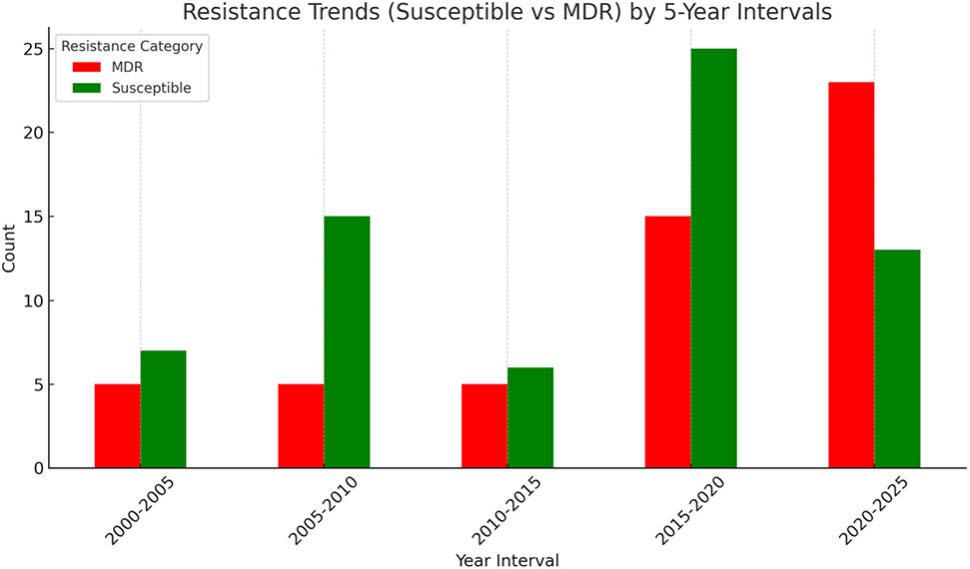
Resistance trends by 5 year intervals MDR - Multidrug Resistance

**Methods:**

We set out to analyze the epidemiology, microbiological profiles, patient demographics, and treatment trends of culture-positive CNS infections over a 25-year period at University of Illinois Health (UIH). We conducted a retrospective analysis of CNS infections identified by positive cerebrospinal fluid (CSF) cultures from 2000 to 2024 at UIH.

**Results:**

Among the 184 positive CSF cultures, 106 cases were identified as nosocomial cases (80% of cases with known etiology). Gram-positive organisms accounted for 97 cases (52%). An increase in multidrug-resistant (MDR) organisms notably increased from 13 (37%) cases in 2015-2019 to 22 total cases (63%) in 2020-2024. Extended-spectrum β-lactamase producing organisms increased from 1 to 3 cases (2.5% to 8.3%) and vancomycin-resistant Enterococcus increased from 0 to 2 cases (0% to 5.5%).

**Conclusion:**

Our 25-year analysis highlights shifting epidemiological trends since 2020, characterized by a notable rise in MDR pathogens, particularly in nosocomial CNS infections. These findings underscore the need for continuous local and national antimicrobial surveillance to guide empirical therapy. Further research is essential to alter guidelines aiming to enhance clinical outcomes and effectively manage emerging AMR infections.

**Disclosures:**

Scott Borgetti, MD, GlaxoSmithKline: Grant/Research Support

